# *Plasmodium knowlesi* gametocyte carriage and load among malaria patients at Kapit Hospital in Sarawak, Malaysian Borneo

**DOI:** 10.1038/s41598-025-32529-2

**Published:** 2025-12-22

**Authors:** Darvindran Theivindran, Ting Huey Hu, Dayang Shuaisah Awang Mohamad, Nawal Rosli, King Ching Hii, Balbir Singh, Angela Siner

**Affiliations:** 1 Malaria Research Centre, Faculty of Medicine and Health Sciences, Universiti Malaysia Sarawak, 94300 Kota Samarahan, Sarawak Malaysia; 2 Ministry of Health Malaysia, Kapit Hospital, 96800 Kapit, Sarawak Malaysia

**Keywords:** Malaria, Medical research

## Abstract

The number of human infections with *Plasmodium knowlesi*, a malaria parasite typically found in long-tailed and pig-tailed macaques, have increased and *P. knowlesi* has become the sole cause of indigenous cases of malaria in Malaysia since 2018. The reasons for the increase are multifactorial and could include human-to-human transmission through mosquito bites. Such transmission would require viable gametocytes circulating in the blood of infected individuals as this is the only parasite blood stage transmissible to mosquitoes. The objectives of this study were to determine the proportion of *P. knowlesi* malaria patients with viable gametocytes and to determine the association between gametocyte load and duration of illness prior to hospital admission, and with total parasitaemia. The mRNA transcripts of *pks25*, a gene expressed in mature female *P. knowlesi* gametocytes, were measured by a real-time PCR assay in blood samples from 295 patients at Kapit Hospital, Sarawak, Malaysian Borneo with PCR-confirmed single infections of *P. knowlesi*. Viable gametocytes were present in 67.5% (199/295) of patients. A positive correlation was seen between gametocyte load and total parasitaemia (ρ = 0.32, *p* = 0.01), whereas there was no statistically significant association between gametocyte carriage and duration of illness prior to hospitalisation (ρ = 0.28, *p* = 0.7). Forty (20%) of 199 gametocyte-positive samples had fewer than 500 *pks25* transcript copies/µL, but 25 (12.5%) of 199 gametocyte-positive patients had elevated levels of gametocytes; 13 (10.8%) had between 10,001 and 100,000 and 12 (6%) had > 100,000 *pks25* transcript copies/µL. Our findings demonstrate the presence of viable gametocytes in a substantial proportion of patients, including some with relatively high densities. This observation, taken together with other findings, underscores the potential of humans to serve as infectious hosts of *P. knowlesi*, but they do not constitute direct evidence of human-to-human transmission. Significant gaps still remain in our understanding of *P. knowlesi* gametocyte biology and infectivity. Addressing these gaps is essential to ascertain whether human-to-human transmission of *P. knowlesi*, which was experimentally demonstrated in the 1960s, occurs in natural settings. Continued surveillance of human *P. knowlesi* infections together with studies on gametocyte biology, vector bionomics, and monitoring of macaque host populations in relation to environmental alterations are vital to understand changes in the dynamics of *P. knowlesi* malaria transmission, and to inform strategies for its control and prevention.

## Introduction

 Malaria is a deadly tropical disease that remains to be the leading cause of morbidity and mortality in endemic countries, with an estimated 263 million cases and 597,000 deaths in 2023^1^. Substantial progress has been made in the last decade in reducing the global burden of malaria and in some countries malaria has been eliminated^1,2^. In Malaysia, the number of human indigenous malaria cases caused by the human malaria parasites *P. falciparum*, *P. vivax*, *P. malariae* and *P. ovale* have seen a drastic reduction in the past few years^3^. However, zoonotic malaria cases caused by *P. knowlesi*, first reported in 2004 as the major causative agent of malaria in the Kapit division of Sarawak, Malaysian Borneo, have become a public health concern in Malaysia^4^. Human infections by *P. knowlesi*, typically found in long-tailed and pig-tailed macaques, have been reported throughout Southeast Asia^5,6^. More recently, human infections due to other simian malaria parasites from these macaques, including *P. cynomolgi*, *P. inui*, *P. coatneyi*, *P. inui*-like, *P. fieldi* and *P. simiovale*, have also been described in Malaysia^7^ and Thailand^8^.

Prior to the initiation of collection of blood samples for the current study in 2016, the number of reported *P. knowlesi* malaria cases were increasing in Malaysia, particularly in the Malaysian Borneo states of Sabah and Sarawak; from less than 500 annual cases of *P. knowlesi* malaria before 2008, to between 848 and 1,487 cases from 2010 to 2016^9^. Knowlesi malaria cases have continued to rise in Malaysia, where from 2018 to 2024 there were 18,965 cases, with *P. knowlesi* being the sole cause of indigenous cases^1,9^. The reasons for this increase in Malaysia are multifactorial including increased awareness of *P. knowlesi* malaria, environmental changes leading to an increase in the population of vectors capable of transmitting *P. knowlesi*, changes in the composition and feeding behaviours of vectors, and land use alterations resulting in loss of habitats of the macaque hosts thereby leading to macaques moving closer to human habitation^10–13^. Another contributing factor towards the increase in zoonotic malaria cases could be human-to-human transmission through mosquito bites. Such transmission of *P. knowlesi* was shown under experimental conditions in the 1960s when the ‘H’ strain of *P. knowlesi*, was transmitted by *Anopheles balabacensis* from one infected individual to 7 others^14,15^. Human-to-human transmission would require viable gametocytes circulating in the blood of infected individuals as this is the only blood stage transmissible to mosquitoes. In the mosquito vector, the gametocytes taken up during a blood meal undergo further development, fertilization and multiplication, leading to the presence in the salivary glands of sporozoites within 10 days^16^.

Gametocytes can be identified by examination of blood films under the microscope but unlike gametocytes of *P. falciparum* which are banana-shaped and morphologically distinguishable from the round trophozoite stages, the gametocytes of *P. knowlesi* are round like the late trophozoite stages, and it is difficult to distinguish between these stages in blood films^17^. Furthermore, when total parasitaemia is low, it is preferable to use molecular detection assays which have been found to be more sensitive than microscopy in studies on gametocyte carriage in individuals infected with *P. falciparum*,* P. vivax* and *P. knowlesi*^18–22^. There are a number of gametocyte-specific genes which are transcribed during different stages of gametocyte maturation for *P. falciparum*; *pfs16* is expressed during the earliest gametocyte stage, *pfpeg3* and *pfpeg4* in stage II gametocytes and *pfs25* in mature stage IV gametocytes^21^. The presence of these gametocyte-specific mRNA transcripts has enabled the development of real-time PCR assays for quantitative gametocyte detection^23,24^. Another advantage of molecular detection methods over microscopy is that they are able to detect and quantify viable gametocytes. Unlike DNA, which can persist in non-viable or lysed parasites, mRNA is rapidly degraded once the parasite dies, making it a more reliable indicator of metabolically active and viable gametocytes. The detection of gametocyte-specific mRNA serves as a functional proxy for the presence of live gametocytes that are capable of fertilization when taken up by a mosquito during a blood meal.

Molecular detection assays for *P. knowlesi* gametocytes have been designed to detect *pks25* which is the orthologue of *P. falciparum pfs25*. Using this assay, Grigg et al. have shown that *P. knowlesi* gametocytes were present in 85% of 100 patients tested on admission to 3 district hospitals in Sabah, Malaysian Borneo^25^. With a different assay, targeting *pks25*, Maeno et al. found that 47% of 32 *P. knowlesi*-infected but asymptomatic individuals from communities living near the forest fringes in Vietnam were also gametocyte-positive. Furthermore, they found 70% of mosquitoes carrying *P. knowlesi* also carried human malaria parasites, thereby supporting the possibility that these mosquitoes became infected with *P. knowlesi* while feeding on humans^26^. In both studies, the time when *P. knowlesi* gametocytes start to appear in the circulation during the course of illness was not reported. The presence of gametocytes in the peripheral blood of individuals in sufficient density early in the infection or before they are treated, would increase the chances of human-to-human transmission, and this could be a contributing factor to the increase in *P. knowlesi* malaria cases. Therefore, a study was undertaken at Kapit Hospital to determine the prevalence of gametocytaemic patients and whether there was any association between gametocyte load with duration of illness prior to hospital admission and with total parasitaemia.

## Results

### Baseline characteristics

**Fig. 1:**
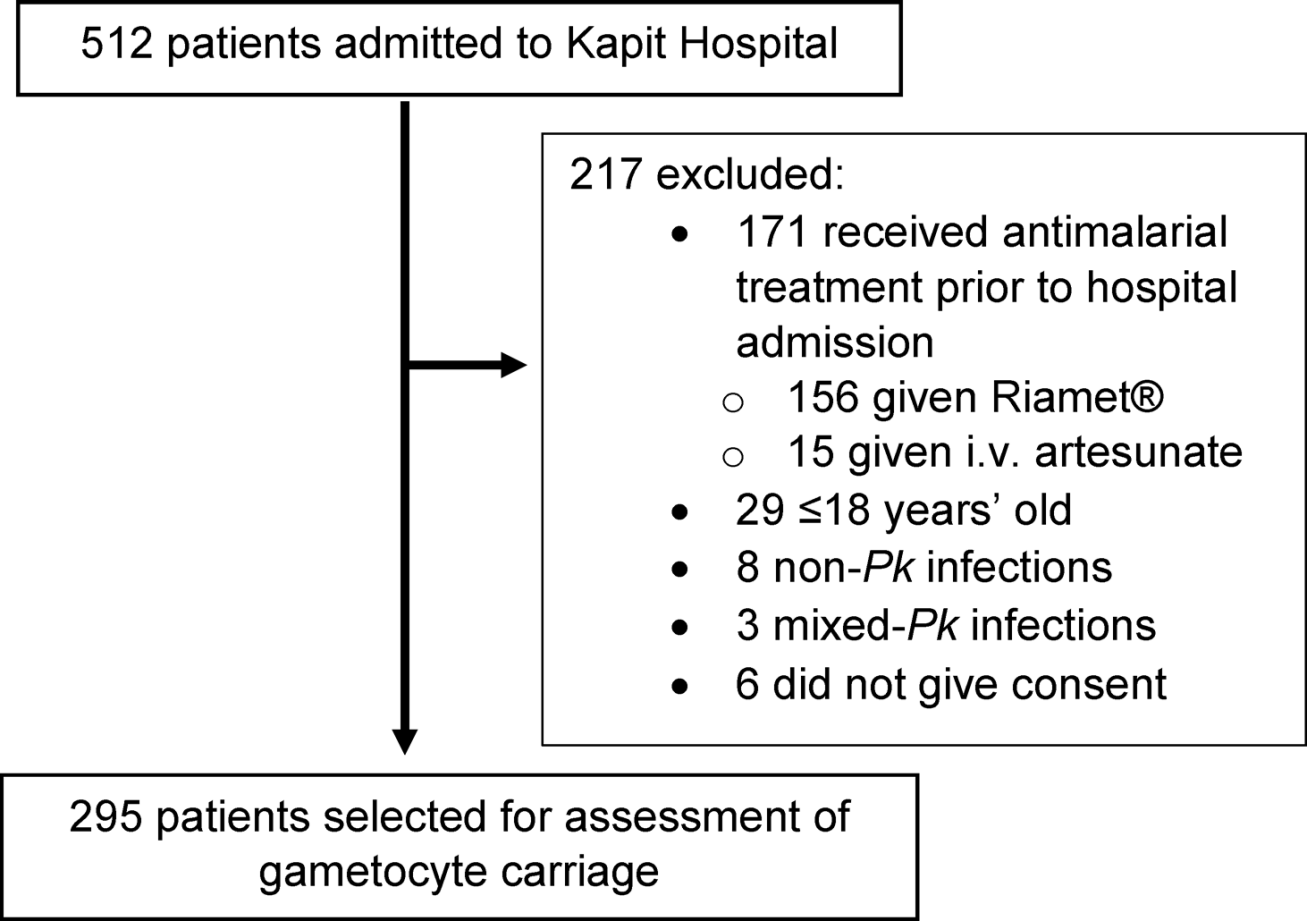
Flowchart of patients selected for gametocyte screening. i.v. = intravenous; *Pk* = *P. knowlesi*.

Of the 512 patients admitted to Kapit Hospital with a diagnosis of malaria, 295 were PCR-confirmed *P. knowlesi* mono-infections (Fig. 1). The included participants were 60% males (*n* = 176) and 40% females (*n* = 119). Mean age of participants was 45 years (range: 18–94 years, SD = 17 years) while median total parasitaemia was 1,829 parasites/µL of blood (IQR: 404–7,989 parasites/µL).

### Gametocyte prevalence and effect of duration of illness on gametocyte carriage, gametocyte load and total parasitaemia

From the 295 patients studied, 199 (67.5%) were gametocyte-positive, and they reported being ill from as early as 2 days, and for one patient as long as 19 days, prior to hospital admission (Table 1). No patients reported being ill for 11 to 13 days or 17 to 18 days. When the relationship between gametocyte carriage and duration of illness was analysed, a Spearman’s rho of 0.28 was observed, which was not statistically significant (*p* = 0.7). Similarly, there was no significant correlation between gametocytaemia and age (Spearman ρ = 0.079, *p* = 0.167), and between parasitaemia and duration of illness (Spearman ρ = 0.034, *p* = 0.95).

Gametocyte load was quantified by measuring the number of transcript copies of *pks25*, a gene transcribed in mature female gametocytes. Quantification values (Cq) ranged between 12.7 and 42.4, and transcript quantities were averaged across replicates and rounded to the nearest Cq value. Forty (20%) of 199 gametocyte-positive samples had fewer than 500 *pks25* transcript copies/µL, but 25 (12.5%) had elevated levels of gametocytes: 13 (10.8%) had between 10,001 and 100,000 and 12 (6%) had > 100,000 transcript copies/µL (Table 1). Only two samples (1%) were found to have more than 1,000,000 *pks25* transcript copies/µL and they were from patients with parasitaemia of 23,754 and 3,798 parasites/µL blood, who had been ill for 2 and 5 days prior to hospital admission.

**Table 1:** Gametocyte carriage and load in relation to duration of illness and total parasitaemia.

Duration of illness (days)	No. of patients	Median parasitaemia^a^ (range: IQR) or parasitaemia	No. gametocyte-positive (%)	Median parasitaemia (range: IQR) or parasitaemia of gametocyte-positive patients^a^	Median gametocyte load^b^ (range: IQR) or gametocyte load of gametocyte-positive patients
2–4	176	3,464 (32–194,505: 348-7,135)	118 (67)	4,551 (66–194,505:1,402–11,788)	1.96 (1–1,460,000: 1.58–21.3)
5–7	104	1,800 (66–293,290: 476-8,546)	73 (70)	4,169 (66–293,290:1,225–13,151)	3.37 (1–828,000: 1.44–125.5)
8–10	8	2,598 (29–102,277: 410–18,258)	5 (62.5)	5,762 (1,774–02,277: 2,507–55,748)	5.34 (1–219,000: 1.97–911)
14–16	2	5,185 & 1,888	2 (100)	5,185 & 1,888	1 & 19.6
19	1	49,298	1 (100)	49,298	56.1

^a^Total number of parasites (asexual stages and gametocytes)/µL blood.

^b^Number of pks25 transcript copies/µL.

Only 33 (32%) of 101 patients with total parasitaemia of less than 500 parasites/µL blood were gametocyte-positive, whereas ≥ 91% of those with parasite counts above 1,000 parasites/µL blood harboured gametocytes and ≥ 93% with parasitaemia above 10,000 parasites/µL were gametocyte-positive (Table 2).

**Table 2:** Total parasitaemia in relation to gametocyte carriage.

Parasitaemia (parasites /µL blood)	Number of patients	Number Gametocyte-positive (%)
< 500	101	33 (32.6)
> 500-1,000	35	19 (54.3)
> 1,000–5,000	68	62 (91)
> 5,000–10,000	34	31 (91)
> 10,000–50,000	33	31 (93.9)
> 50,000-100,000	9	9 (100)
> 100,000	15	14 (93.3)

**Fig. 2:**
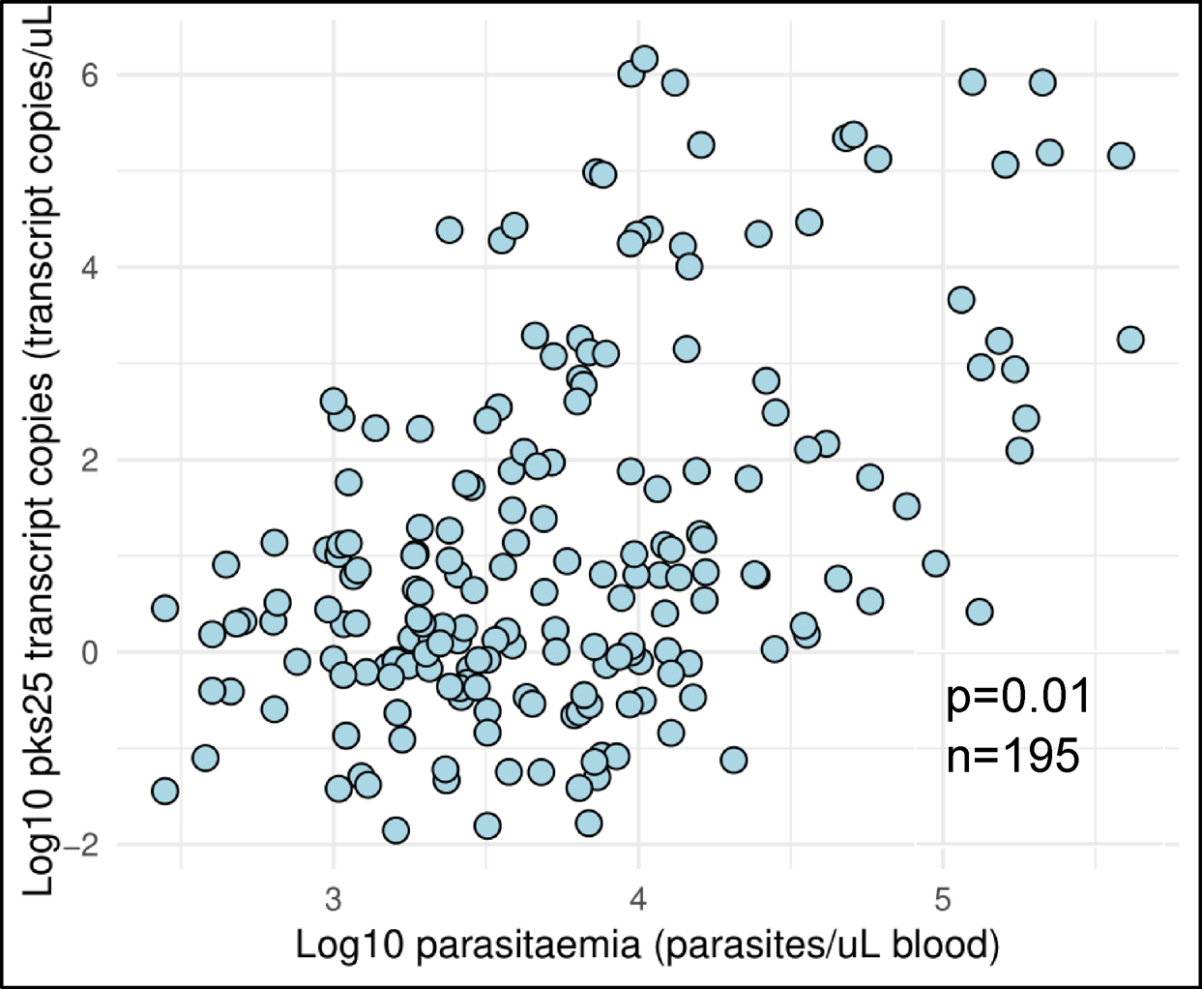
Association between parasitaemia and gametocyte carriage. Spearman’s rank correlation coefficient (ρ = 0.32) that is statistically significant (*p* = 0.01) was observed between parasitaemia and gametocyte load.

A weakly positive Spearman’s rank correlation coefficient (ρ = 0.32) that is statistically significant (*p* = 0.01) was observed between parasitaemia and gametocyte load (Fig. 2).

## Discussion

Overall, viable gametocytes were detected in 67.5% of 295 adult *P. knowlesi* patients on admission at Kapit Hospital, Malaysian Borneo. The gametocyte prevalence observed in our study was lower than the 85% reported by Grigg et al. for 100 patients studied in Sabah, the other Malaysian state in Borneo^25^, and higher than the 47% observed in 32 *P. knowlesi*-positive persons in Vietnam^26^. We used the same *pks25* assay as Grigg et al.; however, differences in the kits used for RNA and cDNA preparation, in the reagents used in the RT-PCR and differences in RNA extraction methods (manual vs. automated) between the studies may have influenced the sensitivity of detection. While our data did not identify duration of illness as a predictor of gametocytaemia and the study in Sabah did not stratify gametocyte carriage according to duration of illness, we cannot exclude those differences in relation to duration of illness may have contributed to the difference in proportion of patients with gametocytes. Another reason for variation in gametocyte prevalence could be differences in the ages of the patients studied. Although we did not find a significant correlation between gametocytaemia and age, higher prevalence was reported in those younger than 20 years of age for *P. falciparum* and *P. vivax*^27–29^. Our samples were collected during a study aimed at comparing clinical presentation in adult patients (> 18 years) while in the study in Sabah, 8.8% of the 226 were aged 12 or younger^25^. With regards to the higher prevalence of gametocyte carriage in our study compared with the Vietnamese study, all our patients had microscopy-positive *P. knowlesi* infections, while all subjects in the Vietnamese study had sub-microscopic asymptomatic infections, with relatively low parasitaemia. Previous studies noted lower detection of gametocytes in sub-microscopic *P. falciparum* and *P. vivax* infections^27,29^ and in our study, total parasitaemia was associated with gametocyte carriage. Therefore, differences in parasitaemia between the two studies could be one of the reasons for the observed differences in gametocyte prevalence.

Gametocytes are the only parasite stages that can continue their development in the mosquito. After a blood meal, they develop into male and female gametes that fertilize to form oocysts. Following sporozoite development in the oocyst, a proportion of these sporozoites invade the salivary glands, making the mosquito infective^16^. The presence of viable gametocytes in our patients does not prove that they are infectious to mosquitoes since there are critical determinants of mosquito infectivity, including gametocyte densities and the male-to-female ratio of gametocytes in the blood. For *P. falciparum*, membrane feeding studies have shown that densities as low as ~ 1 gametocyte/µL can result in mosquito infection, with infectivity increasing steeply with increased gametocyte load before plateauing^30^. These findings underscore that even low-level gametocyte densities, which are often present in asymptomatic carriers, can pose a risk of transmission of falciparum malaria, but there is no similar data for knowlesi malaria. In a recent study in Thailand, membrane feeding experiments with blood from a patient showed that sporozoites were detected in the salivary glands of laboratory-reared *An. dirus* mosquitoes. The parasitaemia of the patient was not stated but of 210 parasites observed, 6 (2.8%) were identified as gametocytes by microscopy^22^. In addition, the experimental studies in the 1960’s using the H strain of *P. knowlesi* demonstrated that *An. balabacensis* mosquitoes fed on a human with 500 parasites/µL blood could transmit the infection to 5 other humans, after each subject received only between 1 and 9 infective bites^14^. No details were provided of the other two humans who were successfully infected following bites from these mosquitoes. A limitation of our study is that we determined gametocyte load as copies of transcripts of *pfs25*, a gene transcribed in mature female gametocytes, but did not convert these to gametocyte counts. Gametocytes can be identified by microscopy but correct identification of *P. knowlesi* gametocytes is challenging, particularly in thick blood films, and results in an underestimation of the actual number of gametocytes. The greater sensitivity of detection of *P. vivax* and *P. falciparum* gametocytes by RT-PCR assays compared with microscopy is well established. In addition, for *P. knowlesi*, Grigg et al. reported that microscopy could detect gametocytes in only 16 (18.8%) of the 85 patients who were gametocyte-positive by RT-PCR. Further work is necessary to determine the minimal gametocyte density of *P. knowlesi* required for mosquito infectivity as this threshold remains unknown. This will require undertaking membrane feeding assays, ideally complemented by direct skin feeding on knowlesi malaria patients, with local vectors of *P. knowlesi* in Malaysian Borneo, such as *An. balabacensis* and *An. latens*^31,32^. Application of dose-response modelling, as has been utilised for *P. falciparum*, would enable estimation of transmission threshold for *P. knowlesi*. Another factor which plays a key role in determining success of malaria transmission from humans and other hosts to mosquitoes is the gametocyte sex ratio. The RT-PCR assay used in *P. knowlesi* studies detects transcripts of *pks25*, expressed by mature female gametocytes^33^. Therefore, assays designed to detect mature male gametocytes need to be developed and the gametocyte sex ratio in human *P. knowlesi* infections needs to be quantified. This data will be essential for assessing transmission risks.

A positive correlation was observed in our study between gametocyte load and total parasitaemia in knowlesi malaria patients at clinical presentation. Although the parasitaemia in knowlesi malaria patients is generally low, *P. knowlesi* infections can lead to high parasite counts. At Kapit Hospital, Sarawak and in 3 district hospitals in Sabah, 11.2% (47/420) and 14.6% (64/437) of knowlesi malaria patients had admission parasitaemia of > 20,000 parasites/ µL blood respectively^34,35^. Patients with higher parasitaemia would be expected to carry a greater number of gametocytes compared to those with low parasitaemia since maturation of *P. knowlesi* gametocytes is faster than the 8–12 days observed for *P. falciparum*^36^. Gametocytes of *P. knowlesi*, a species with a short erythrocytic cycle of 24 h, have been reported to mature within 1.5 to 2 days^36,37^, and may be detected within 3 days of a heavy inoculum of infected blood in monkeys^37^. Experimental infections with macaques suggest that mature *P. knowlesi* gametocytes are likely to persist in circulation for only a short period but this needs to confirmed in human infections^38^. Interestingly, despite relatively high total parasitaemia (> 1,000 parasites/µL of blood), 12 patients in our study were gametocyte-negative. The transcription factor APETALA2 (ApiAP2) is a master regulator of gametocytogenesis in *Plasmodium* species, with its expression levels influencing gametocyte production in *P. falciparum*^39–41^. However, the role of this transcription factor in *P. knowlesi* gametocytogenesis remains uncharacterised. Investigating the molecular mechanisms regulated by APETALA2 in *P. knowlesi* may not only elucidate the factors contributing to the absence of gametocytes but also shed light on the conditions that promote their development leading to high gametocyte levels.

Individuals with high gametocyte loads are at greater risk of transmitting *P. knowlesi* to new hosts through mosquito vectors, particularly if they participate in outdoor activities and postpone seeking treatment at health facilities. People living in close proximity to clinical cases have been reported with low-density, asymptomatic *P. knowlesi* infections^42^. While a modelling-based study of over 30,000 malaria cases, including over 23,000 knowlesi malaria cases reported in Malaysia did not find any evidence supporting sustained non-zoonotic *P. knowlesi* transmission, such an analysis based on surveillance data of patients at hospitals from 2012 to 2020 would not have been able to detect a relatively small number of cases resulting from human-to-human transmission in areas where the transmission is primarily zoonotic^43^. Critically, while human-to-human transmission is possible, its contribution to the overall number of human knowlesi malaria cases is likely to be small compared to infections acquired from non-human primate reservoir hosts. This disparity is fundamentally linked to mosquito feeding behaviour and the only comparative entomological study to date found that *An. latens* in the Kapit district of Sarawak feeds preferentially on macaques^44^. Consequently, the modelling-based analysis cannot prove that human-to-human transmission by mosquitoes is not occurring^53^, but it indicates that such a transmission is not sustained. Although human-to-human transmission was demonstrated with *An. balabacensis*, one of the vectors of knowlesi malaria in Sabah and northern Sarawak, under experimental conditions in the 1960s proving that this occurs in nature would be a major challenge since all the human knowlesi malaria infections take place in areas where humans and macaques live in close proximity^14,45,46^. Even if molecular characterisation of *P. knowlesi* from a cluster of patients within a community over a short time frame identifies identical haplotypes, it will be impossible to determine whether these parasites originated from macaques or humans. A deeper understanding of *P. knowlesi* gametocyte biology, particularly the quantification of gametocyte sex ratios and thresholds in human infections required for mosquito infectivity is critically needed. Such information is essential to more accurately evaluate the potential and relative frequency of mosquito-mediated human-to-human transmission of *P. knowlesi* in natural settings.

In conclusion, the findings of our study, taken together with those of previous studies, underscores the potential of humans to serve as infectious hosts for *P. knowlesi*. However, significant gaps remain in our understanding of *P. knowlesi* gametocyte biology and infectivity. Addressing these gaps is essential to ascertain whether human-to-human transmission of *P. knowlesi* occurs in natural settings. Continued surveillance of knowlesi malaria is necessary since ecological changes, that includes changes in land use due to agricultural needs, loss of habitat of the macaque hosts, human population expansion, combined with alterations in the feeding behaviour of mosquitoes, could potentially allow *P. knowlesi* and other simian malaria parasites to switch host. In addition, a decrease in the macaque population could drive *P. knowlesi* parasites to adapt and circulate within the human population. Thus, continued surveillance of human *P. knowlesi* infections, together with detailed studies on gametocyte biology, vector bionomics, and monitoring of macaque host populations in relation to environmental alterations is vital to understand changes in the dynamics of *P. knowlesi* malaria transmission, and to inform strategies for its control and prevention.

## Methods

### Study area and population

Kapit Division, one of the twelve administrative divisions in Sarawak, Malaysian Borneo has a total population of 134,800, is served by 16 health clinics and one district hospital called Kapit Hospital^47^. This division was selected as the study site due to the high incidence of *P. knowlesi* malaria cases that have been reported for the past decade couple with infected individuals living in longhouses that are close to the forests.

Samples from this study site were collected during a study which compared the clinical, laboratory and epidemiological features of two subpopulations of *P. knowlesi*^34^. Samples were collected from November 2016 to October 2018, consisting of patients who presented to Kapit Hospital. Inclusion criteria were adults with PCR-confirmed single *P. knowlesi* infections and who had not taken antimalarial drugs prior to hospital admission.

Ethical clearance for this study was obtained from the Medical Research and Ethics Committee of the Ministry of Health Malaysia (NMRR-16-943-31224(IIR)) and the Medical Research Ethics Committee of Universiti Malaysia Sarawak (UNIMAS/NC-21.02/03 − 02 Jld. 2 (19)). All methods were performed in accordance with the relevant guidelines and regulations in accordance with the Declaration of Helsinki.

### Blood collection, sample storage and transport

Whole blood samples were taken on hospital admission from each patient after informed consent was obtained. Aliquots of 50 µL were mixed with 250 µL of RNAprotect^®^ (Qiagen, Germany). Duplicates were made for each sample and tubes were labelled accordingly with sample ID, patient initials and date. Samples were stored at -80 °C at Kapit Hospital until further collection by staff of the Malaria Research Centre (MRC) at Universiti Malaysia Sarawak. Samples were shipped frozen to the MRC in a liquid nitrogen dry shipper (Core Cryolab, Canada) and stored immediately in -80 °C upon arrival.

### Parasite count and species confirmation

Thick and thin blood films were prepared at Kapit Hospital using finger-prick blood samples. At MRC, thick blood films were stained by Giemsa and used to determine parasite counts. Parasite counts were determined using the average readings of two independent experienced microscopists and were derived from the number of parasites (both asexual and sexual stages) per 500 white blood cells, adjusted by the total white blood cell count for each patient. DNA was extracted from the blood spots prepared using InstaGene™ (Bio-Rad, USA) as described previously^48^. Nested PCR assays for identification of malaria species with species-specific primers primers for *P. falciparum*,* P. malariae*,* P. vivax*,* P. ovale*,* P. knowlesi*,* P. cynomolgi* and *P. inui* were employed as described before^49^. Only patients with a PCR-confirmed *P. knowlesi* mono-infection were included in this study.

### Molecular analysis

Total RNA was extracted on the QIAcube automated system (Qiagen, Germany) using the RNeasy Mini Kit protocol (Qiagen, Germany), and eluted in 30µL of Tris-EDTA (TE) buffer. On-column DNA digestion was also included during the process using the RNase-free DNase set (Qiagen, Germany). Extracted total RNA was then reverse-transcribed to cDNA using the SuperScript™ VILO™ cDNA synthesis kit (Invitrogen™, USA) in a total volume of 20 µL that consisted of 7 µL nuclease-free water, 4 µL 5X VILO™ reaction mix, 2 µL 10X SuperScript^®^ enzyme mix and 7 µL RNA. The no-reverse transcriptase control (-RT) was also prepared for each sample, where the 10X SuperScript^®^ enzyme mix was omitted from the master mix. This is to enable a check for genomic DNA contamination. The reaction mix was incubated at 25 °C for 10 min, followed by at 42 °C for 60 min and terminated by incubation at 85 °C for 5 min. Synthesis of cDNA was verified by amplifying cDNA templates targeting *Pk* serine tRNA ligase sequences using previously published primers^50,51^. Briefly, PCR reactions were performed in a total volume of 20 µL, consisting of 10 ng of cDNA template, 7.9 µL nuclease-free water, 1× Green GoTaq^®^ Buffer (Promega, USA), 1.5 mM MgCl₂, 800 µM dNTP mix (Promega, USA), 0.1 µM each of forward and reverse primers, and 1.25 U GoTaq^®^ DNA Polymerase (Promega, USA). The thermocycler parameters were set as follows: 1 cycle of initial denaturation (94 °C, 10 min); 35 cycles of denaturation (94 °C, 30 s), annealing (55 °C, 30 s) and extension (72 °C, 1 min); 1 cycle of final extension (72 °C, 7 min). The standard curve method was employed for absolute quantification of *pks25*, a gametocyte-specific gene expressed in mature female gametocytes. Quantification of *pks25* was conducted using a TaqMan^®^ assay with previously published probe and primers on Mastercycler^®^ ep realplex (Eppendorf, Germany)^25^. Plasmid DNA containing the amplicon sequence were used as standards. Standard curves were generated by performing 10-fold serial dilutions starting at 1,000,000 copies/µL to 1 copy/µL. The limit of detection (LOD) was determined using this standard curve and determined to be ~ 1.4 copies/µL, based on the lowest dilution consistently detected across replicate runs. Amplification efficiency and linear range were calculated in each qPCR run. The Fit Points method was used for absolute quantification^52^. Unknown sample concentrations were extrapolated to the standard curve generated. Negative controls were also included in triplicates. Quantities of the *pks25* transcripts were reported as the average transcript numbers of the replicates to the nearest quantification cycle (Cq) value.

### Statistical analysis

Clinical and molecular data were recorded in Microsoft Excel (Redmond, WA: Microsoft). Prior to statistical analyses, the number of *pks25* transcript copies and total parasitaemia were log transformed to the log base of 10 (log10). Statistical analyses were performed using R Statistical Software (v4.1.2; R Core Team 2021) with data visualization conducted using the ggplot2 package (v3.3.3; Wickham, 2016). Variables were tested for normality using the Shapiro-Wilk test and the Spearman’s rank correlation was used to test for relationship between the two non-normal log transformed variables.

## Data Availability

The datasets generated during and/or analysed during the current study are available from the corresponding author on reasonable request.
